# Environmental Behavior Spillover or Public Information Induction: Consumers’ Intention to Pay a Premium for Rice Grown with Green Manure as Crop Fertilizer

**DOI:** 10.3390/foods10061285

**Published:** 2021-06-04

**Authors:** Fuduo Li, Kangjie Zhang, Aibo Hao, Changbin Yin, Guosheng Wu

**Affiliations:** 1 Institute of Agricultural Resources and Regional Planning, Chinese Academy of Agricultural Sciences, Beijing 100081, China; lifuduo@caas.cn (F.L.); 18874362808@163.com (K.Z.); haoaibo163@163.com (A.H.); 2 Research Center for Agricultural Green Development in China, Beijing 100081, China

**Keywords:** intention to pay a premium, pro-environmental behavior, pro-environmental food, environmental behavior spillover, public information induction, rice grown with green manure as crop fertilizer (GMR)

## Abstract

Nowadays, there is a growing interest in pro-environmental foods produced by pro-environmental practices. However, consumers’ payment motivations towards such foods are currently poorly understood. This manuscript provided a critical investigation of Chinese consumers’ intention to pay a premium (ITPP) for rice grown with green manure as crop fertilizer (GMR). One focus was the establishment of an explanatory structural research framework that includes effects of environmental behavior spillover (EBS) and public information induction (PII); another focus was to analyze the impacts of the selected structural elements on ITPP by introducing education as a moderator. Results suggest that consumers’ ITPP can be largely influenced by PII, therefore, for GMR marketers and policy makers, measures should be developed to widen consumers’ access to public information related to GMR and to improve their capacity of screening effective information. EBS, when ITPP remains low, emerged as a pivotal predictor of consumers’ ITPP. This observation provides us with the enlightenment that breeding consumers’ daily environmental behaviors is highly valued to inspire their payment intention in the early stages of GMR market development. Another finding is that, with the introduction of the educational variable, the influence coefficients of EBS and PII on ITPP increased from 0.42 and 0.53 to 0.61 and 0.66, respectively, which means that it is possible to boost consumers’ payment intention by improving their educational attainment. This study contributes to the existing literature by providing empirical evidence for the GMR industrial upgrading strategy and have significant implications for the environmental governance of the agricultural sector.

## 1. Introduction

The use of chemicals in agriculture has strongly contributed to the increased food outputs observed over the last few decades. However, intensive inputs of these industrial synthetics have led to severe environmental consequences and widespread food safety concerns. As a response, an improved agricultural practice, green manure as substitute for chemical fertilizers, was promoted in southern China with government encouragement. Previous studies have fully explained the ecological service functions of green manure crops. They believe that green manure can not only replace chemical fertilizers to provide nutrients for crops, but also contribute to increasing soil organic matter, retarding soil erosion and passivating soil heavy metals, thus improving the total environment of the soil and the safety of agricultural products [1,2]. Obviously, the sustainable development and utilization of green manure is of vital importance to safeguarding the ecological security and promoting high-quality agriculture in China.

The sustainability development of green manure essentially depends on how farmers benefit from foods grown with green manure as crop fertilizer (GMFs), especially under a low government subsidy for the planting of this crop, at present. As noted by Mbwaga et al., planting green manure will make the farming system more complex and labor-intensive, which will inevitably lead to higher production costs [3]. In this case, higher returns from GMFs are essential to stimulate producers to maintain their planting behavior. In a market economy, the income of producers is determined by the price of goods and the price of goods is closely related to consumers’ payment intentions. Although intermediaries (processors, marketers, etc.) may extract some of the value of goods in market transactions, the degree to which consumers pay is still the most pivotal aspect that determines the income of producers. Therefore, a better understanding of how consumers are paying for GMFs is crucial for both environmental policy makers and participants in the GMF industry.

Unfortunately, consumers’ payment reactions towards GMFs are currently poorly understood, which is why this study was conducted initially. Previous studies have proven that consumers are generally willing to pay premiums for pro-environmental foods (ENFs) (ENFs refer to foods produced using pro-environmental techniques or methods; GMFs belong to the category of ENFs) and that such payments tend to incentivize producers to act consistently in a pro-environmental way. For instance, Wu et al. pointed out that consumers paying a premium for environmental pork attributes can incentivize farmers to adopt cleaner production technologies in pig production [4]. Li et al. revealed that if consumers were willing to pay a premium for environmental beef, nomads would also be more inclined to adopt climate-friendly practices [5]. In terms of labeled rice, Yu et al. stated that consumers paying a premium for “Green rice” affect farmers’ behavioral intention to adopt environmental-friendly technologies [6]. Zhou et al. emphasized that farmers who could benefit from the eco-labeled rice were more likely to adopt environmental behavior [7]. GMFs provide long-term benefits to both the environment and humanity based on its emphasis on safety, nutrition and environmental friendliness. As consumers have realistic demands for products with these attributes, it is theoretically possible for them to pay premiums for GMFs. Despite the rapid development of the GMF industry over the past few years, a mature market has not yet been established. Consumers’ perceptions and purchase intentions of this product have not been clearly identified. Will consumers actually have any intention to purchase GMFs at higher price (vs. conventional foods) and what factors will ultimately influence their purchase decisions? A comprehensive understanding of these issues will help policy makers both in the agriculture and consumer sectors to make more precise policy responses.

Existing literature on consumer paying premiums for ENFs has generally paid attention to explain how observable socioeconomic characteristics influence their purchasing decisions. These influencing factors include demographic information, such as respondents’ gender, age, education level, health condition, etc. [8], socioeconomic characteristics, such as their family size and population structure, income, dietary habit, etc. [9], and various perceptions of the food attributes and the external environment [10]. These variables provide simplified representations of the potential drivers involved in given contexts [11]. However, the simplified index system is always accompanied by insufficient explanatory information, which makes it challenging to unearth the deeper incentives for consumer behavior. Under this circumstance, several research paradigms considering more meta-factors have been developed, most of which emphasized the impact of “green consumerism” [12,13] and “information technology” [14,15].

Studies conducted by de Leeuw et al. found that pro-environmental consumers are more likely to pay premiums for ENFs [16]; meanwhile, Grunert et al. believed that consumers’ intention to pay a premium (ITPP) for the given ENF was mainly due to information intervention from public channels [17]. Actually, consumers’ initial understanding of emerging ENFs either comes from the spillover of the green awareness that already exists with the same underlying process or from the inducible intervention of external information [18]. Thus, the effects of environmental behavior spillover (EBS) and public information induction (PII) may be the underlying factors driving consumers to purchase ENFs, such as GMFs. Although an increasing attention has been paid to EBS and PII by scholars [7], those studies have not empirically demonstrated the specific path of their impact on consumers’ ITPP. Moreover, the environmental awareness related to EBS and the information screening related to PII are closely associated with the education of consumers. What role does education play in the mechanisms by which EBS and PII affect ITPP? The lack of discussion on these critical topics hinders the improvement of policy makers’ awareness and the formulation of consumption policies related to GMFs.

In China, rice is the most important food crop, both in terms of production and consumption. In recent years, the Chinese government has implemented a series of supporting policies, such as subsidy policy for rice growers and minimum purchase price policy, which have achieved comprehensive support for rice farmers in multiple links and at multiple levels. According to the China Statistical Yearbook, in 2018, the proportion of rice sown area in the total coverage of grain crops of China was 30.5% and the proportion of rice yield in China’s grain output was 34.8%; both ratios are higher than those of other grain crops. Therefore, improvements in rice-based farming modes provide the broadest support for agro-environmental governance. In fact, in the last few years, the production of pro-environmental rice has been strongly supported by the government and the domestic market of these types of rice has experienced rapid development (Table 1). Rice grown with green manure as crop fertilizer (GMR), in particular, has achieved a more significant increase in planting area and yield, compared with rice that requires professional certification, such as green rice and organic rice. China has nearly 13 million hectares of cultivated field suitable for the production of GMR and the current output of GMR is still less than 20% of the potential production capacity. If these production capacities are fully released, it will have a major impact on China’s food industry and agricultural environment. We thus decided to choose GMR as the representative of GMFs (or ENFs) to carry out this study.

The overall aim of this study is to develop a novel research framework containing EBS and PII to explore consumers’ ITPP for GMR. Specifically, this paper aims to make progress in the following two aspects: (1) To determine the path and extent of the impact of EBS and PII on consumers’ ITPP. Considering that consumers’ education can influence their environmental cognition and information identification, this factor will be included in the research framework as a moderating variable. (2) To reveal the influence mechanism of education on consumers’ ITPP. It is understood that there have been few previous studies on consumers’ ITPP for GMR and our exploration is probably one of the cutting-edge ones. The expected findings of this paper can be provided to policy makers as an opportunity to gain insight into the factors that influence consumer preferences for GMR.

The remainder of this paper is organized into five sections: Section 2 sets up the theoretical framework and puts forward the research hypothesis. A detailed introduction to GMR is also included in this section. Section 3 introduces the study area, survey design, data collection and research methods. Section 4 shows the empirical results and section five discusses the main findings and puts forward the research deficiencies. The final section sums up the research conclusions.

## 2. Theoretical Framework and Hypotheses Development

### 2.1. Description of GMR: Production Process and Attributes

**Figure 1:**
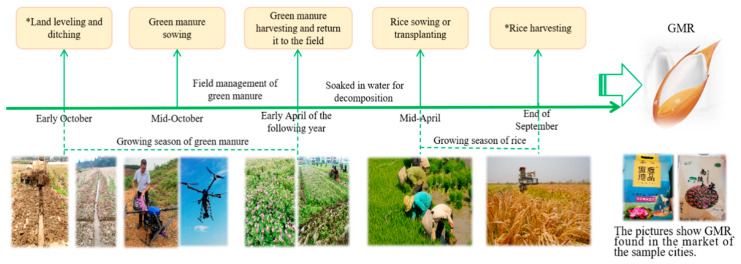
The process of GMR production.

The production process of GMR can be described as follows: green manure crops are sown in fallow fields that have been leveled and lined with drainage ditches in early October. Subsequently, refined field management is carried out after seeds have germinated, including taking measures to prevent livestock from trampling; in early April of the following year, green manure is mechanically returned to the fields, then soaked in water for a week or two for decomposition. The rice-growing season runs from mid-April to the end of September. Figure 1 shows the critical nodes of GMR production. It can be seen that, compared with conventional rice production, the production process of GMR is more complex, requiring more input elements and higher production cost.

Rice that meets the following conditions simultaneously can be certified as GMR. On the one hand, the nutrients needed for the growth of rice crops are provided by green manure instead of chemical fertilizers. On the other hand, the nutritional value and safety of the products comply with the established standards. Based on this, we can conclude three attributes of GMR: environmental friendliness, safety and nutrition. The production of GMR requires the whole process to be carried out without fertilizer input. Therefore, it has less impact on the environment and less chemical residue in agricultural products. A report from China Green Manure Research System (2019) shows that the total amino acid content and essential amino acid content in GMR were 120% and 155% higher than those of conventional rice. This highly nutritious rice is more in line with current consumer needs. More importantly, the better taste of GMR highly meets the “hedonistic” preferences of urban consumers.

### 2.2. The Connotation of EBS and PII

#### 2.2.1. EBS

The concept of EBS was first proposed by Thøgersen, who deemed that one environmental behavior can be catalyzed by other environmental behaviors with the same underlying process in specific context [19]. Since then, a growing number of literatures have emerged to interpret the connotation of EBS in diversified angles. For example, Thøgersen and Crompton pointed out that people who implement certain target environmental behaviors will also be more willing to perform more non-targeted environmental behaviors in other areas [20]. Truelove et al. defined EBS as an influence of an intervention on subsequent environmental behaviors not targeted by the intervention [21]. Penz et al. argued that EBS referring to past environmental behavior increases the likelihood of subsequent environmental behavior [22]. Though these studies give slightly different descriptions of what EBS means, they are essentially the same, that is, EBS is a phenomenon whereby an individual’s target environmental behavior catalyzes the non-targeted environmental behavior based on the same underlying ideology.

Consistent with the above literature, we focused on the positive sense of EBS, in our case, the attempt to encourage consumers to transform their daily environmental behaviors into pro-environmental intention of paying premiums for ENFs. For city dwellers, they have more or less adopted pro-environmental practices, either because of their environmental awareness or for the sake of saving living costs (for instance saving water and electricity resources). Sustainable environmental behavior will eventually catalyze a “green lifestyle” that further induces residents to be “green consumerists”. Actually, there also exists a negative spillover effect discussed in previous studies [23,24]. These studies indicate that a first environmental behavior does not necessarily catalyze a second and even hinders the generation and reinforcement of other environment behaviors. However, such negative spillover occurs only when the successful increase in one environmental behavior is associated with a reduction in another [21] and are therefore outside the focus of the present research.

#### 2.2.2. PII

In information economics, public information can also be defined as social information, which refers to the information from external channels that individuals or groups are exposed to in social activities [25,26]. Previous studies on public information mainly focused on two aspects: sources and functions. Regarding the source, media advertising [27,28], government release [29] and interpersonal interaction [30] are the three main channels that the existing research paid more attention to. Especially in recent years, with the increasing diversification of media means, information from new media platforms (such as WeChat and TikTok) has attracted more and more attention. Individuals exposed to public information for a long time are likely to be induced into consistent behavior [31]. This induction effect has been found to play a role in farmers’ adoption of green technology in the production field [32] and consumer payment for high-quality commodities in the consumption field [33]. Accordingly, PII is often used as a policy tool to regulate the markets of production and consumption to ensure the stable operation of the macro economy.

### 2.3. The Impact of EBS and PII on Consumers’ ITPP for GMR

#### 2.3.1. The Influence of EBS on ITPP

The EBS proposes that engaging in one behavior affects the probability of engagement or disengaging in a second behavior [34]. Many studies have demonstrated the existence of EBS. For example, Truelove et al. explored the positive and negative spillover of environmental behavior by identifying different decision modes as competing mechanisms that drive the adoption of initial environmental behaviors [21]. Albornoz et al. examined the effect of the role of spillovers on the environmental actions of Argentinean firms [35]. Ek and Miliute-Plepiene found that behavioral spillovers of environmental policy are present in the collection of food waste in Sweden [36]. EBS is derived from the impact of “catalyst behaviors” of environmental awareness [7]. Environmental behaviors that are in line with consumers’ current lifestyle might catalyze basal shifts to more complicated ones such as purchasing ENFs due to similar underlying ideologies [37]. According to Nilsson et al., the positive spillover effect predicts that interventions targeting one specific environmental behavior have the capacity to promote non-targeted and/or future environmental behaviors [34]. Therefore, the following hypothesis was proposed:

**Hypothesis** **1** **(H1).** *The stronger the EBS, the more likely consumers are to pay a premium for GMR.*

#### 2.3.2. The Influence of PII on ITPP

PII originates from the “induced consciousness” generated by external information intervention [38]. When the respondents received the information intervention, one could specifically capture the effect of “induced consciousness” on ENFs preferences [39]. Information intervention increases information exposure and information exposure multiplies the ways in which consumers are influenced by the actions of social public subjects, including people around them, media and government [40]. According to Latacz-Lohmann and Foster, information from these social subjects significantly improved their payment attitude to ENFs and further resulted in conscious payment behavioral intentions [41]. Given this logic, the following hypothesis was proposed:

**Hypothesis** **2** **(H2).** *The stronger the PII, the more likely consumers are to pay a premium for GMR.*

#### 2.3.3. Differences in the Impact between PII and EBS

As for the emerging ENFs, which lack stable consumer preferences, EBS and PII have significantly different influence mechanisms on consumer behavioral intention in paying for such foods. In general, the influence of EBS on consumer payment intention tends to be greater than that of PII, when the ITPP remains low. The reason is that the cognition of emerging foods is currently lacking, for consumers. Their initial purchasing preference, although influenced by public propaganda, will not be determined by it. The tentative psychology from the underlying ideology of pro-environment may be more powerful in determining the intention of consumers [42,43]. With the enhancement of ITPP and the improvement of consumers’ trust in ENFs, the impact of PII on intentions is increasing due to the directness of its role [44]. Accordingly, the following hypothesis was proposed:

**Hypothesis** **3** **(H3).** *There are conditional differences in the impact of EBS and PII on consumers’ ITPP.*

#### 2.3.4. The Moderating Effect of Education

Individuals’ environmental behaviors can be positively affected by education. Generally speaking, the higher the level of education, the stronger their environmental awareness and the more likely environmental behaviors will occur and catalyze EBS [45]. On the other hand, the extent to which individuals pick up public information and the resulting inductive behaviors may also be affected by their education level. Education increases the opportunity for individuals to be exposed to information and enhances their ability to identify and capture effective information [14]. In fact, some leading research has focused on the positive impact of education on individuals’ behavioral intentions by regulating EBS or PII, but there is still a lack of empirical exploration to integrate the three components into a unified research framework [46]. Our research will make a breakthrough on this point and proposes the following hypothesis:

**Hypothesis** **4** **(H4).** *Education has a favorable impact on consumers’ ITPP by positively moderating EBS.*

**Hypothesis** **5** **(H5).** *Education has a favorable impact on consumers’ ITPP by positively moderating PII.*

#### 2.3.5. Socio–Demographic Factors as Control Variables

Socio–demographic factors were the most basic dimension of influencing factors of consumers’ ITPP. In particular, previous studies suggest a prominent role of gender, age, household size and household income, in determining consumers’ ITPP for ENFs. For example, Klopi et al. focused on the impact of gender and age on consumers’ ITPP for nutrition and health claims on food products [47]. Chekima et al. paid attention to the influence of gender, age and yearly income on consumers’ ITPP for organic food [48]. Grasso and Asioli further explored the impact of household size on consumer purchasing intention and behavior of upcycled ingredients, such as biscuits [49]. Accordingly, our study includes gender, age, household size and household income as the control variables.

### 2.4. Research Framework

Several studies about ENFs have developed the diversified research frameworks on the determinants of ITPP, each of which was tested by structural models to investigate its statistical significance. In this study, the original theoretical framework explored the impact mechanisms of EBS and PII on ITPP. Subsequently, a moderating variable—education—was added into the original framework to make the framework more explanatory.

**Figure 2:**
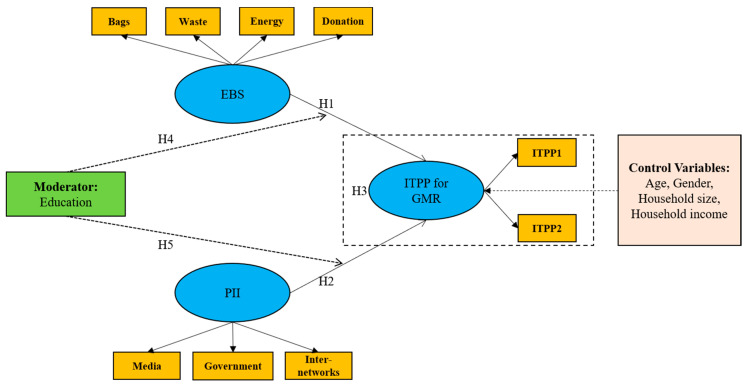
The theoretical framework for determinants of consumers’ ITPP for GMR.

To clarify the influence of EBS on consumers’ ITPP for GMR, four typical environmental behaviors, namely, using degradable plastic bags, sorting domestic waste, saving energy or resources and donating to environmental organizations, were considered, based on the literature [50,51,52]. It should be noticed, however, that whether these behaviors “catalyze” consumers’ ITPP depends on the consistency of the underlying motivations, which can differ across behaviors [53,54]. For using degradable plastic bags, donating to environmental organizations and sorting domestic waste, environmental motivations are more critical than non-environmental motivations, while for saving energy or resources, both significant environmental and non-environmental motivations exist. Therefore, different spillovers were expected from different environmental behaviors. Meanwhile, when exploring the influence of PII on consumers’ ITPP for GMR, public information from three main sources, including media advertising, government public releases and interpersonal networks, were taken into account. The analysis framework of this paper is shown in Figure 2.

## 3. Material and Methodology

### 3.1. Study Area

At present, GMR is mainly produced and consumed in the southern rice region (SRR) of China. Especially in Hunan, Hubei, Jiangxi and Anhui, the output of GMR in the four provinces accounted for about 60% of the national total in 2018 [55]. Considering that GMR is mainly provided to local residents, these four provinces are also the main consumption areas of GMR. Therefore, the four provinces in SRR were selected as the representatives of this study. Given that the consumers of GMR are mainly urban residents, especially the consumers in big cities, the provincial capital of Changsha (Hunan province), Wuhan (Hubei province), Nanchang (Jiangxi province) and Hefei (Anhui province) were therefore chosen to conduct our survey. The four provincial capitals are the largest cities in their respective provinces, both in terms of population and economic size [56]. Research on consumers’ ITPP for GMR in these cities can provide key information for consumption policy making.

### 3.2. Procedure and Samples

#### 3.2.1. Questionnaire and Pilot

The survey design was primarily in accordance with the theoretical framework shown in Figure 2. The initial questionnaire consisted of five parts, i.e., respondents’ socio-demographic characteristics, conventional environmental behaviors, exposure to the public information related to GMR, awareness and availability/accessibility of GMR, as well as the ITPP for GMR. After completing the initial questionnaire, a professional group, consisting of one professor, two associate professors and six doctoral students, was summoned to check the items to enable them to be understood easily. To further improve the quality of the questionnaire, a pilot survey was conducted in Changsha in mid-November 2019 and 152 sample data were obtained. According to the pilot data analysis, the unreliable and invalid items in the original questionnaire were eliminated. We ultimately obtained the official version of the questionnaire, including 37 indicators in 5 sets, which can be used for the formal survey.

#### 3.2.2. Survey

**Figure 3:**
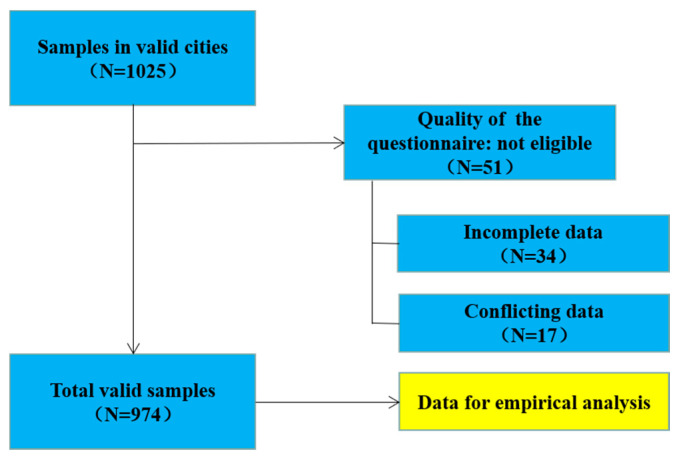
Online sample screening process.

The formal survey was conducted online. At the end of November 2019, the official version of the questionnaire was submitted to Questionnaire Star, a professional online survey platform maintaining a giant customer base of around 6.5 million people in mainland China [57]. To ensure that the respondents come from the target cities and minimize the selection bias, sample requirements were set by establishing strict screening procedures. Furthermore, the paid sample service of the platform was chosen and the respondents who successfully finished the survey were rewarded with certain quotas. The detailed data collection and screening processes are shown in Figure 3. A total of 1025 respondents passed the preliminary screening question set by the platform. However, 51 invalid questionnaires were excluded due to incomplete or contradictory data during the second round of system screening. Finally, 974 effective online questionnaires, including 234 from Wuhan, 248 from Nanchang, 272 from Changsha and 220 from Hefei, were obtained.

#### 3.2.3. Reasonableness of Sample Size

According to Wang et al., when the number of potential respondents is enormous, there is no necessary relationship between the minimum sample size available for study and the total population [58]. In this case, it is only affected by the error and confidence level. The formula for calculating the minimum sample size is shown in Equation (1) and the results are shown in Table 2.

(1) $$n=Z^{2}\sigma^{2}/d^{2}$$

where n denotes the sample size. Z represents the statistics under a certain level of confidence. σ is the standard deviation of the population, set to 0.5. d is the allowable error and is designed as a 50% confidence level in this study.

Generally speaking, a confidence level of 90% and an allowable error of 3% are appropriate for the samples. We can thus confirm that the minimum sample size is 752, which verifies that our sampling survey meets the theoretical requirements.

### 3.3. Methodology

#### 3.3.1. Reliability and Validity Analysis

Cronbach’s α was used to evaluate the validity and reliability of the questionnaire results. Validity refers to the correctness of the measurement. The higher the validity, the more the measurement results can reflect the real characteristics of the content to be measured. Reliability is the consistency and stability of measurement results. The larger the measurement error, the lower the measurement reliability will be [59]. Generally speaking, it can be considered that the analyzed data had a good internal consistency when Cronbach’s α was greater than 0.6. Subsequently, an exploratory factor analysis (EFA) was carried out to check the factorability and suitability, by using the measurement parameter of Kaiser-Meyer-Olkin (KOM) and Bartlett’ s test of sphericity, respectively.

#### 3.3.2. Structural Equation Model (SEM)

Given that this study mainly investigates the relationship between consumers’ ITPP and EBS and PII, many latent variables are included; the linear specification estimated by ordinary least squares regression may be biased, when evaluating the factors that determine consumers’ ITPP. Therefore, the structural equation model (SEM) was applied for theoretical modeling and results analysis in our study.

According to Anderson and Gerbing [60], SEM can be defined as follows:

(i) the structural model:

(2) η=γξ+βη+ζ

where ξ represents the standardized exogenous latent variables and η represents the endogenous latent variables. β and γ are parameters to be estimated and represent the effect coefficients of the interaction between the endogenous latent variables and the effect coefficient of the influence of exogenous latent variables, respectively. ζ is the residual vector of η.

(ii) the measurement model:

(3) Y=λyη+ε

(4) X=λxξ+δ

where Y and X represent the vectors of endogenous and exogenous observable variables. λy represents the relationship between endogenous latent variables and its observable variables. λx represents the relationship between exogenous latent variables and its observable variables. ε and δ are the residual matrix of the measurement model.

## 4. Results

### 4.1. Descriptive Information

#### 4.1.1. Socio-Demographic Characteristics of the Respondents

The socio-demographic characteristics of the surveyed consumers involve their gender, age, education, health status, social identity attributes, household size, household income and many other aspects. Considering that our study mainly focuses on the correlation between consumers’ ITPP and EBS and PII, these factors, that need to be considered as control variables, were simplified. As mentioned above, we selected only the socio-demographic factors that have been proved by most of the previous studies to have a significant impact on consumer behavioral intentions, including gender, age, household size and household income, for description and statistical analysis. As shown in Table 3, among the 974 respondents, gender distribution was relatively balanced, with a slight majority of female participants. Most of the respondents were between the ages of 31 and 40 with a high level of education. A large proportion of the respondents had four or five members in their families, while the average annual household incomes were between 10 × 10^4^ and 20 × 10^4^ CNY. The results of Cramer’s V showed that no significant differences existed in these control variables between the four cities.

#### 4.1.2. The Measured Items

Indicators involved in this theoretical framework were latent variables, which need to be measured by the corresponding observable variables. Specifically, consumers’ environmental behaviors were reflected in the frequency with which they adopt specific environmental items. Similarly, the impact of public information was represented by the frequency with which consumers pick up external interventions. Consumers’ responses to EBS and PII were measured by the 3-point Likert-type scale from “Never” to “Always or often”. Consumers’ ITPP was reflected by their responses to “‘intent’ and ‘plan’ to pay a premium for GMR”. An overview of the latent variables is shown in Table 4.

**Figure 4:**
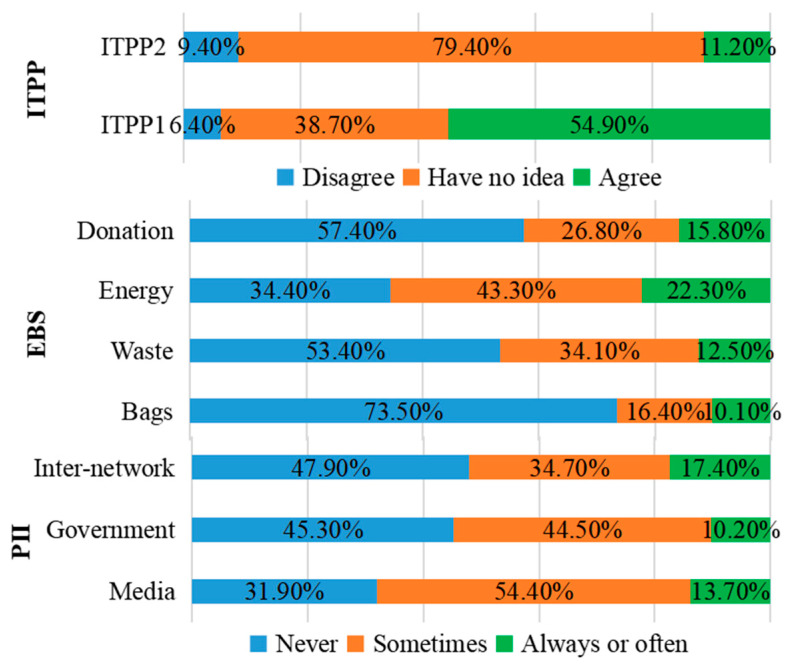
Descriptive information of observable variables.

**Figure 5:**
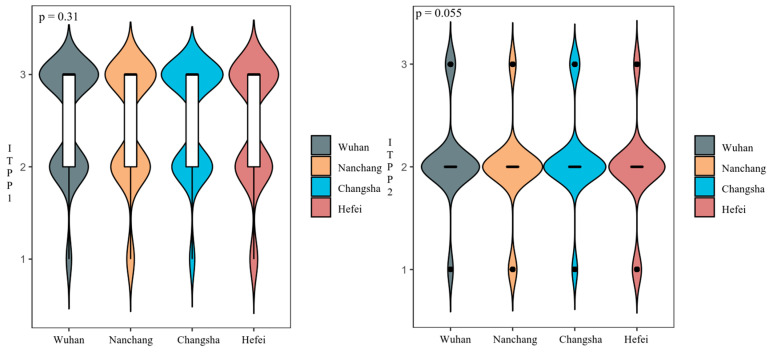
Results of homogeneity of variance test (95% CI).

According to Figure 4, approximately 54.8% of the surveyed consumers responded positively to ITPP1, compared with 11.2% for ITPP2. This indicated that most consumers are willing to try to purchase GMR at a premium, but in the long run, there is uncertainty about their purchasing preference. Results of the homogeneity of variance test showed that no significant difference existed for neither ITPP1 nor ITPP2 among the four cities (Figure 5), hence the regional dummy variable will not be considered in the economic modeling in this study.

With regard to environmental behavior, consumers responded most positively to the adoption of energy-saving measures compared with the other three practices. This may be due to the fact that energy-saving is a cost-cutting practice [61]. Consumers are the most cautious about using biodegradable shopping bags, which may be because such high-cost environmental behaviors increase their household expenditures and aggravate their financial burdens [62]. Under the current sluggish growth in wage income of urban residents, this will undoubtedly worsen the welfare of consumers. As for the impact of public information, consumers are most affected by the interpersonal network composed of the interactions with the people around them, followed by the influence of media advertisements, while the influence of government public propaganda is relatively small. How can the government play a better role in guiding consumers to purchase GMR? The expected conclusions of this study may be useful for solving this problem.

### 4.2. Measurement Model

Table 4 presents the results of EFA. The Cronbach’s α of each construct was >0.7 and the standard factor loading of each observable variable was also >0.6. Meanwhile, the overall Cronbach’s α was 0.897, which indicates that the latent variables can be well reflected by the observable variables that were selected in our study and the model is reliable enough to be used for analysis. In addition, the KMO = 0.666 and the *p* value of Bartlett’s test of sphericity was 0.000, which verifies the applicability of factor analysis.

### 4.3. Structural Model

#### 4.3.1. Goodness of Fit

Based on Hair et al., it is necessary to evaluate the fitness of the theoretical structure model for the observable variables before using SEM [63]. In this study, 3 categories of 11 indices were selected [64] to assess the fitness and the results are shown in Table 5. From what can be seen, all indices are better than or close to the recommended levels. Therefore, the survey data are suitable to be analyzed by this hypothetical model.

#### 4.3.2. Results of SEM

**Figure 6:**
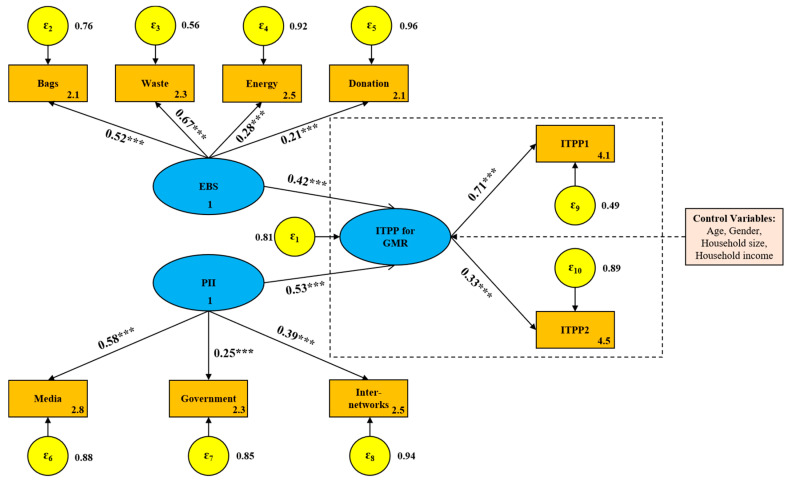
SPCs of the original structural model. Note: “***”, significant 1% level.

**Figure 7:**
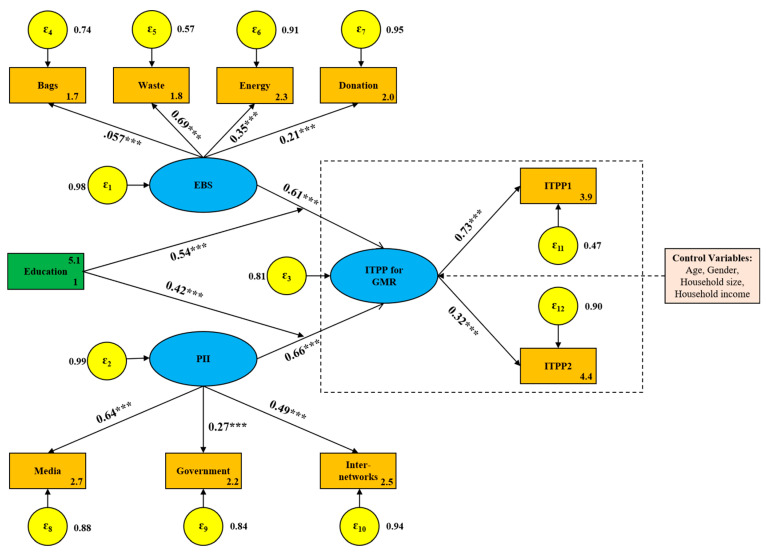
SPCs of the SEM with moderator. Note: the moderator, education, was also measured by the 3-point Likert-type scale from “Low” to “High”. “***”, significant 1% level.

**Figure 8:**
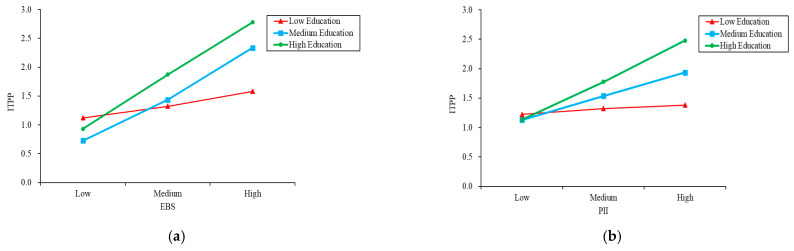
Graphical representation of moderating effects. Note: (**a**) the moderating effect of education on the association of EBS with ITPP; (**b**) the moderating effect of education on the association of PII with ITPP.

The standardized path coefficient (SPC) of the original SEM is shown in Figure 6. As can be seen from the estimation results, both EBS and PII had a significant positive effect on ITPP. PII had a larger impact on consumers’ ITPP, with a SPC of 0.53 (*p* < 0.01), while EBS had a relatively little influence, with a SPC of 0.42 (*p* < 0.01). In Figure 7, a moderator, education, was introduced into the SEM, which enhances the influence degree of EBS and PII on consumers’ ITPP. Although PII still had a greater impact on ITPP than EBS, the path coefficient from EBS to ITPP increased by 45.2%, compared with 24.5% for PII. This suggests that consumers’ intention to buy GMR can be more powerfully catalyzed by enhancing their environmental behaviors through improved education. The moderating effect of education on the association of EBS and PII with ITPP is illustrated in Figure 8.

The relations between latent variables and observable variables can also be obtained from the path analysis in Figure 6 and Figure 7. On the whole, the observable variables in the two models have a consistent response relationship to the corresponding latent variables. EBS was mostly reflected by consumers’ behavior of sorting household waste, followed by the use of biodegradable plastic bags and saving energy or resources in daily life, while consumers’ donation behavior to environmental organizations had a relatively weak impact on EBS. PII was mainly revealed by media advertising, followed by the influence of interpersonal interactions, while the factor regarding government induction had a weak explanatory ability for PII. Similarly, consumers’ ITPP was significantly reflected by the immediacy intention rather than the long-term plan to pay a premium for GMR, indicating that the preference of consumers for GMR still stems from their tentative psychology.

#### 4.3.3. Hypothesis Testing

According to the results of the two models, all hypotheses are fully verified. First, both EBS and PII had significant positive effects on ITPP, which confirmed **H1** and **H2** Second, the influence of PII on ITPP was significantly higher than that of EBS and **H3** was verified. Third, compared with the original SEM estimation, the SPCs of the influence of EBS and PII on ITPP were significantly increased in the improved SEM containing the moderating factor, indicating that **H4** and **H5** were supported. The detailed results of hypothesis testing are shown in Table 6.

### 4.4. Diagnostic Analysis: Further Examine the Impact of EBS and PII on ITPP

**Figure 9:**
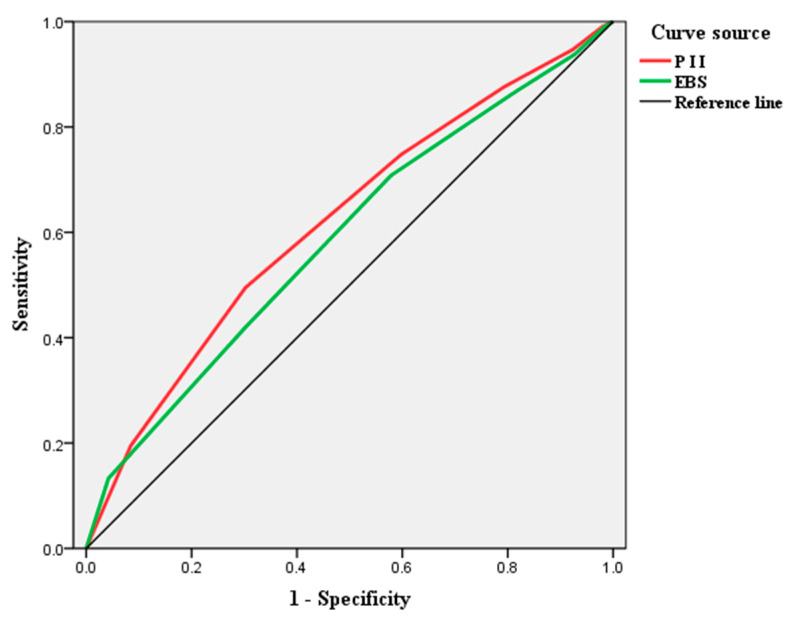
Diagnostic analysis of consumers’ ITPP to EBS and PII changes.

Before the diagnostic analysis, EBS, PII and ITPP were standardized, respectively, by taking the weighted mean values of their respective observable variables containing them. In this survey, consumers’ responses to questions related to ITPP were measured by a 3-point Likert-type scale. Only when the respondents fully approved the statements in the questionnaire and chose the “Agree” option, we confirmed that they have the intention to pay a premium for GMR. Accordingly, the median value of ITPP can be used as a criterion. Consumer responses higher than the median value were defined as “High” and the responses less than, or equal, to the median value were defined as “Low”. Subsequently, a diagnostic analysis was performed to examine the responsiveness of consumer ITPP to changes in EBS and PII. According to Figure 9 and Table 7, ITPP is more responsive to changes in EBS when consumers have less intention to pay a premium for GMR. However, as ITPP increases, its response to changes in PII becomes more and more obvious. This indicated that the impact of EBS and PII on consumers’ ITPP is heterogeneous in time series, which further supported **H3**.

## 5. Discussions and Implications

This study has enhanced the understanding of what characterizes consumers’ emerging environmental foods payment intention in China. It is among the first studies developing a conceptual model to understand how EBS and PII, together with the moderating effects of education, work together in determining consumers’ ITPP for GMR. Therefore, the findings will be valuable for policy makers who are promoting sustainable consumption of this product in their effort to improve the welfare of consumers. In addition, producers and marketers of GMR should also consider the findings of this research while drafting more effective strategies to ensure more consumption of their product at a higher level of payment.

The findings of this study confirmed that PII is, on the whole, the most salient factor affecting consumers’ ITPP for GMR, which is consistent with some studies about consumers’ ITPP for high-quality foods in emerging markets. For example, in China, Loebnitz and Aschemann-Witzel [65] showed that external information intervention enhanced consumers’ awareness of organic food and thus their willingness to buy. In Vietnam, Schöll et al. found that the information intervention projects significantly increased consumer interest in environmental pork [66]. In India, Singh and Verma pointed out that public information inducement was an important factor in determining consumers’ purchase of organic food products [67]. In Malaysia, Ahmad et al. also stated that public information reinforced consumer behavior in paying for rice attributes [68]. The possible reason is that, in developing countries, the ENFs market emerges late and develops slowly, consumers’ understanding of this category of product is insufficient and stable consumption preference has not yet been formed. In this case, consumers’ ITPP for GMR is more likely to be influenced by external induced information.

The above result provides implications for the marketing of GMR. Since positive information effectively guide consumers to pay a premium for GMR, public inducement policies can be developed to promote the industrialization of such products. First, our findings suggested that information intervention from media advertising was the most important aspect reflecting PII. Hereby, TV, internet and other novel media tools can be used to disseminate GMR-related information to enhance consumers’ willingness to pay for GMR. The better effect of media information on inducing consumers may be related to its high exposure [69]. In addition, media information has another feature, visibility, which can also help to promote the enhancement of consumers’ behavioral intention [7]. This is because visual information is usually presented in words, pictures and other more trustworthy ways rather than in auditory forms, such as audio broadcasts, and is therefore more impressive, in line with the connotation implied by the Chinese proverb “Hearing is empty while seeing is believing”. Second, information intervention from interpersonal networks was considered to be another vital factor revealing PII, which provides us with the enlightenment that it is of great significance to build a trust-based interpersonal interaction mechanism to promote consumers to purchase GMR at a premium. Dirks argued that interpersonal trust is highest among acquaintances [70]. Therefore, more attention should be paid to GMR-related information diffusion through the channels of acquaintance interaction, especially to GMR marketers. Third, we found a weak impact of direct information released by the government on PII; this does not mean that the government’s role in GMR promotion is not important. The function and effectiveness of media and interpersonal network information channels need the power of the government to guarantee. Hence, how the government can play a better role in channeling public information is an issue that needs to be considered.

Our study also found that consumers’ ITPP for GMR can be significantly affected by EBS, which provides evidence of “catalyst behaviors”. On the surface, environmental behaviors seem to have nothing to do with consumers’ intention to pay for GMR; however, this recognition ignores the fact that any environmental behavior is based on the same underlying individual ideology [71,72]. Purchasing and consuming ENFs is essentially an environmental behavior. The consistency of the underlying process makes it possible for it to be catalyzed by other environmental behaviors, such as sorting domestic waste, using biodegradable shopping bags, saving resources and energy and donating to environmental organizations. The spillover effect of consumers’ environmental behaviors can be generated by two mechanisms: self-identity and consistency. Self-identity derives from self-perception reinforced by initial behaviors, which leads to future behaviors appropriate to the role [73]. Consistency is self-consistent behaviors that prevent the discomforts of cognitive dissonance and conflicting attitudes [74]. With these mechanisms working together, environmental behaviors appropriate for consumers’ current lifestyles catalyzed their intention to pay for GMR. Actually, our survey also supports the findings that EBS exists to a certain extent. Approximately 60% of respondents with the intention of paying a premium for GMR had engaged in at least one of the environmental behaviors in the past year; consumers that perform more environmental acts have a stronger intention to pay.

One novel finding is that EBS enhanced consumers’ ITPP more effectively when the payment intention remained low instead of high, which suggests that the role of EBS should be paid more attention to in the early stage of the rollout of a certain ENF. As an emerging ENF, GMR is rarely found in the market at present and consumers universally have no clear understanding of this product, which leads to their low payment intentions. According to our survey, although nearly 55% of respondents said they would be willing to make a tentative purchase of GMR, only 11% of them have long-term payment plans that can contribute to the industrialization of GMR. This situation fits with the underlying conditions for using the EBS to improve consumer behavior. Specifically, two stimuli can be taken to enhance the effect of EBS [21]. One is to continuously strengthen consumers’ environmental awareness and increase the intensity of their environmental behavior. Both conscious learning and compulsory education are conducive to the improvement of consumers’ environmental awareness [45]. The second is to break through the external barriers of environmental behaviors catalyzing consumers’ ITPP for GMR, such as regulating the market supply and demand of GMR to keep a relatively stable price and ensuring that the safety and nutrition claims of GMR are consistent with the facts.

As expected, education significantly improved the impact of EBS and PII on consumers’ ITPP for GMR. Many studies have recognized the key role of education in improving consumers’ payment intention for ENFs, but little in-depth discussion has been conducted on how it works. In this study, two paths were discovered through which education plays a role. First, education enhances consumers’ environmental awareness by improving their environmental knowledge, which in turn leads to high-intensity environmental behaviors [75,76]. Persistent and frequent environmental behaviors ultimately “catalyze” consumers’ intention to pay for GMR. Second, education positively acts on consumers’ payment intention for GMR by increasing consumers’ information channels and their ability to discriminate effective information [77]. Given the conditions under which EBS and PII play a role, we found that the incentives of education on consumers’ ITPP is consistently effective, regardless of the intensity of ITPP. This finding provides policy makers with the enlightenment that strengthening general education and environmental education of urban residents is of great significance for promoting consumers’ intention to pay for GMR and promoting the rapid industrialization of this emerging ENF.

It is also worth noting that the contribution of this research goes beyond the specific case discussed here. It has other important connotations for scholars and policy makers. First of all, it is one of the few literatures currently exploring the ITPP for emerging ENFs such as GMR. Second, the analytical framework and economic model of this study can be generalized to a great degree and it can be applied to other consumer intentions and behaviors. Moreover, the study results and policy implications of this paper are not only of great value to the industrialization of GMR, but may also be of a certain reference value to the formulation of agricultural environmental policies related to RGRS.

This study provided a new perspective for a comprehensive understanding of Chinese consumers’ ITPP for an emerging ENFs, GMR; however, limitations inevitably exist. On the one hand, the empirical data used in this study came from online surveys, which may be followed by problems associated with sample selectivity bias. In fact, many studies on consumer intentions or behaviors have adopted online surveys based on the most extensive sampling considerations when collecting data. They generally believe that, despite the limitations of sample targeting, selectivity bias can be controlled, as long as the sample size is big enough [49,78,79]. Future surveys will continue to expand the sample size and design more optimized screening procedures to minimize the impact of survey techniques. On the other hand, the four cities examined in this study were provincial capitals in southern China with a relatively large population and a high GDP, while consumer behavior in small- and medium-sized cities was not taken into account. Obviously, people living in cities with different economic statuses have distinguished consumption habits. Therefore, it is necessary for future studies to expand the scope of investigation and pay attention to the differential comparison of consumers’ intentions or behaviors in cities of different sizes.

## 6. Conclusions

The majority of past studies related to consumer intentions mainly focused on the influence of individual or family endowments, market environment, cognitive factors, etc., on their payment decisions and the most commonly used empirical methods were various regression analyses. Through structural modeling, the current research contributes to the existing literature in that it provides evidence for the influence mechanism of the catalytic effect of environmental behavior and inductive effect of public information on consumers’ ITPP. Specifically, we firstly validated the applicability of the established structural framework in the analysis of consumers’ ITPP for GMR. Subsequently, we empirically explored the impact of EBS and PII on consumers’ ITPP and further tested the moderating effects of education on the two factors. According to the results, consumers’ ITPP can be largely influenced by PII; therefore, for GMR marketers and industry policy makers, measures that can broaden consumers’ access to information and improve their capacity for screening effective information should be developed and adopted. EBS, when ITPP is at a low level, emerged as a pivotal independent predictor of consumers’ intention. This observation provides policy makers with the enlightenment that cultivating consumers’ daily environmental behaviors is highly valued to improve their payment intention for the emerging GMR. Another finding is that education significantly improved the positive effects of EBS and PII on ITPP; this result indicates that it is possible to enhance consumers’ payment intention by means of education.

## Figures and Tables

**Table 1:** Comparison of GMR with green rice and organic rice.

		2013			2018			Growth Rate (%)	
	Label	Number of Certified Rice	Certified Area (10^5^ ha)	Total Amount of Rice (10^5^ t)	Number of Certified Rice	Certified Area (10^5^ ha)	Total Amount of Rice (10^5^ t)	Certified Area	Total Amount of Rice
Organic rice	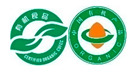	3019	32.83	132.75	4872	39.98	166.79	21.78	25.64
Green rice	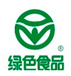	2501	289.45	1162.36	5351	328.26	1558.65	13.41	34.09
GMR	No certification required. The process of growing green manure is subject to government supervision.	——	Planting area 132.45	1058.89	——	Planting area 237.33	1737.48	79.18	64.09

Data source: CGFDC Web site (http://www.greenfood.agri.cn/ztzl/tjnb/lssp/) (accessed on 10 July 2019). CARS Web site (http://www.cars.ren/) (accessed on 22 May 2019).

**Table 2:** The minimum sample size at the different error and confidence levels.

Confidence Level		80%	85%	90%	95%	99%
Allowable error	1.0%	4096	5148	6766	9604	16,590
	2.0%	1024	1296	1692	2401	4148
	3.0%	456	576	752	1068	1844
	4.0%	256	324	423	601	1037
	5.0%	164	208	271	385	664

**Table 3:** The socio-demographic characteristics of the respondents.

Index	Definition	Number of Respondents					Chi-Square Test	
		Total	Changsha	Wuhan	Nanchang	Hefei	(Cramer’s V)	Sig.
Gender	Male	440	122	106	112	100	0.043	0.322
	Female	534	150	128	136	120		
Age	≤30	188	58	50	44	36	0.021	0.156
	31–40	272	74	66	70	62		
	41–50	222	60	52	58	52		
	51–60	176	48	42	46	40		
	>60	116	32	24	30	30		
Education	Junior high school and below (Low)	135	32	20	39	44	0.038	0.227
	High school or advanced vocational education (Medium)	297	83	75	67	72		
	University and above (High)	542	157	139	142	104		
Household size	<4	186	26	58	62	40	0.066	0.734
	4–5	438	130	92	110	106		
	>5	350	116	84	76	74		
Household income (×10^4^ CNY per year)	<10	166	48	34	44	40	0.016	0.512
	10–20	378	110	88	94	86		
	20–30	304	76	80	78	70		
	≥30	126	38	32	32	24		

Note. “CNY” refers to Chinese yuan, 1 CNY = 0.1516 USD (12 November 2020).

**Table 4:** Descriptive statistics of the items.

Latent Variables	Observable Variables	Description	Response Scale (1–3)	Mean	Standard Deviation	Item Loadings	Cronbach’s α for Each Construct
ITPP	ITPP1	I intend to pay a premium for GMR in the future	1 = Disagree, 2 = Have no idea, 3 = Agree	2.485	0.614	0.726	0.669
	ITPP2	I have a long-term plan to pay a premium for GMR		2.016	0.453	0.637	
EBS	Bags	The frequency of using biodegradable plastic bags	1 = Never, 2 = Sometimes, 3 = Always or often	1.366	0.658	0.815	0.734
	Waste	The frequency of sorting domestic solid waste		1.591	0.702	0.722	
	Energy	The frequency of saving water or electricity		1.877	0.743	0.851	
	Donation	The frequency of donating time or money to environmental protection organizations		1.585	0.748	0.679	
PII	Media	The frequency of picking up information about GMR from media advertising	1 = Never, 2 = Sometimes, 3 = Always or often	1.819	0.651	0.834	0.662
	Government	The frequency of picking up information about GMR from the government releases		1.651	0.658	0.796	
	Inter-network	The frequency of picking up information about GMR from interpersonal networks		1.696	0.749	0.842	
Overall Cronbach’s α value			0.897				
Kaiser–Meyer–Olkin (KMO)			0.666				
Bartlett’s test of sphericity			0.000				

**Table 5:** Goodness of fit measures of SEM model.

Fit Index	Index	Recommended Level	Estimate Value for Hypothetical Model	GOF Judgment
Absolute fit indices	χ^2^/df	<2	1.617	Supported
	RMR	<0.05	0.034	Supported
	RMSEA	<0.05	0.036	Supported
	GFI	>0.9	0.896	Close to
	AGFI	>0.9	0.911	Supported
Incremental fit indices	NFI	>0.9	0.925	Supported
	IFI	>0.9	0.897	Close to
	TLI	>0.9	0.913	Supported
	CFI	>0.9	0.902	Supported
Parsimony fit indices	PNFI	>0.5	0.729	Supported
	PGFI	>0.5	0.637	Supported

**Table 6:** Results of the SEM with moderator and hypothesis testing.

Path	Estimate			C.R. ^m^	P ^m^	Hypothesis	Supported
	SPC ^o^	SPC ^m^	Var.				
ITPP ← EBS	0.42	0.61	0.19	7.330	***	H1	YES
ITPP ← PII	0.53	0.66	0.13	5.216	***	H2	YES
(ITPP ← EBS) ← Education		0.54		8.434	***	H4	YES
(ITPP ← PII) ← Education		0.42		7.552	***	H5	YES

Note. SPC ^o^: the standardized path coefficient of the original model; SPC ^m^: the standardized path coefficient of the model with moderator. Var.= SPC ^m^ -SPC ^o^; C.R. ^m^: the critical ratio of the model with moderator under standardized path; “***”, significant at 1% level.

**Table 7:** Test results for diagnostic analysis.

Variable	Overall Sensitivity	S.E. ^a^	Gradual Sig. ^b^	95% CI	
				Low	High
PII	0.619	0.018	0.000	0.584	0.654
EBS	0.589	0.018	0.000	0.553	0.624

Note. ^a^: Nonparametric assumptions; ^b^: Null hypothesis, real sensitivity = 0.5.

## Data Availability

The data presented in this study is available on request from the corresponding author.

## References

[1] Moreau P., Ruiz L., Raimbault T., Vertes F., Cordier M., Gascuel-Odoux C., Masson V., Salmon-Monviola J., Durand P. (2012). Modeling the potential benefits of catch-crop introduction in fodder crop rotations in a Western Europe landscape. Sci. Total Environ..

[2] Knott S.C. (2015). An analysis of the financial implications of different tillage systems within different crop rotations in the Swartland area of the Western Cape, South Africa. Ecol. Econ..

[3] Mbwaga A. (2006). On-farm verification and promotion of green manure for enhancing upland rice productivity on Striga-infested fields in Tanzania. Final Technical Report. Am. J. Ophthalmol..

[4] Wu L., Wang S., Zhu D., Hu W., Wang H. (2015). Chinese consumers’ preferences and willingness to pay for traceable food quality and safety attributes: The case of pork. China Econ. Rev..

[5] Li X., Jensen K., Clark C., Lambert D.M. (2016). Consumer willingness to pay for beef grown using climate friendly production practices. Food Policy.

[6] Yu X., Gao Z., Zeng Y. (2014). Willingness to pay for the “Green Food” in China. Food Policy.

[7] Zhou J., Liu Q., Mao R., Yu X. (2017). Habit spillovers or induced awareness: Willingness to pay for eco-labels of rice in China. Food Policy.

[8] Da Motta R.S., Ortiz R.A. (2018). Costs and Perceptions Conditioning Willingness to Accept Payments for Ecosystem Services in a Brazilian Case. Ecol. Econ..

[9] Malawska A., Topping C.J., Nielsen H.Ø. (2014). Why do we need to integrate farmer decision making and wildlife models for policy evaluation?. Land Use Policy.

[10] Hernández-Espallardo M., Arcas-Lario N., Tantius P.H. Farmers’ satisfaction and intention to continue as members of agricultural marketing co-operatives: A test of the neoclassical and transaction costs theories. Proceedings of the 113th EAAE Seminar “A resilient European food industry and food chain in a challenging world”.

[11] Knowler D., Bradshaw B. (2007). Farmers’ adoption of conservation agriculture: A review and synthesis of recent research. Food Policy.

[12] Moisander J.K. (2010). Motivational complexity of green consumerism. Inter. J. Consum. Stud..

[13] Chekima B., Chekima K., Chekima K. (2019). Understanding factors underlying actual consumption of organic food: The moderating effect of future orientation. Food Qual. Prefer..

[14] Xiang Z., Magnini V.P., Fesenmaier D.R. (2015). Information technology and consumer behavior in travel and tourism: Insights from travel planning using the internet. J. Retail. Consum. Serv..

[15] Vicente-Molina M.A., Fernández-Sáinz A., Izagirre-Olaizola J. (2013). Environmental knowledge and other variables affecting pro-environmental behaviour: Comparison of university students from emerging and advanced countries. J. Clean. Prod..

[16] De Leeuw A., Valois P., Ajzen I., Schmidt P. (2015). Using the theory of planned behavior to identify key beliefs underlying pro-environmental behavior in high-school students: Implications for educational interventions. J. Environ. Psychol..

[17] Grunert S.C., Juhl H.J. (1995). Values, environmental attitudes, and buying of organic foods. J. Econ. Psychol..

[18] Han Z., Zeng D., Li Q., Cheng C., Shi G., Mou Z. (2019). Public willingness to pay and participate in domestic waste management in rural areas of China. Resour. Conserv. Recycl..

[19] Thøgersen J. (1999). Spillover processes in the development of a sustainable consumption pattern. J. Econ. Psychol..

[20] Thøgersen J., Crompton T. (2009). Simple and Painless? The Limitations of Spillover in Environmental Campaigning. J. Consum. Policy.

[21] Truelove H.B., Carrico A., Weber E.U., Raimi K.T., Vandenbergh M.P. (2014). Positive and negative spillover of pro-environmental behavior: An integrative review and theoretical framework. Glob. Environ. Chang..

[22] Penz E., Hartl B., Hofmann E. (2019). Explaining consumer choice of low carbon footprint goods using the behavioral spillover effect in German-speaking countries. J. Clean. Prod..

[23] Riera O., Swinnen J. (2016). Household level spillover effects from biofuels: Evidence from castor in Ethiopia. Food Policy.

[24] Geng L., Chen Y., Ye L., Zhou K., Chen Y. (2019). How to predict future pro-environmental intention? The spillover effect of electricity-saving behavior under environmental and monetary framing. J. Clean. Prod..

[25] Valone T.J., Templeton J.J. (2002). Public information for the assessment of quality: A widespread social phenomenon. Philos. Trans. R. Soc. B Biol. Sci..

[26] Blanchet S., Clobert J., Danchin É. (2010). The role of public information in ecology and conservation: An emphasis on inadvertent social information. Ann. N. Y. Acad. Sci..

[27] Borra S.T., Bouchoux A. (2009). Effects of Science and the Media on Consumer Perceptions about Dietary Sugars. J. Nutr..

[28] Borda D., Mihalache O.A., Dumitraşcu L., Gafițianu D., Nicolau A.I. (2021). Romanian consumers’ food safety knowledge, awareness on certified labelled food and trust in information sources. Food Control.

[29] Meijer A., Thaens M. (2009). Public information strategies: Making government information available to citizens. Inf. Polity.

[30] Holma A.-M. (2012). Interpersonal interaction in business triads—Case studies in corporate travel purchasing. J. Purch. Supply Manag..

[31] Mastrobuoni G. (2011). The role of information for retirement behavior: Evidence based on the stepwise introduction of the Social Security Statement. J. Public Econ..

[32] Kabunga N., Dubois T., Qaim M. (2012). Heterogeneous information exposure and technology adoption: The case of tissue culture bananas in Kenya. Agric. Econ..

[33] Kurihara S., Maruyama A., Shimoura S., Nishiyama M., Luloff A.E., Hirose M., Matsuda T. (2006). Research about the influence of food safety information on consumer behavior. HortResearch.

[34] Nilsson A., Bergquist M., Schultz W.P. (2017). Spillover effects in environmental behaviors, across time and context: A review and research agenda. Environ. Educ. Res..

[35] Albornoz F., Cole M.A., Elliott R.J., Ercolani M.G. (2014). The environmental actions of firms: Examining the role of spillovers, networks and absorptive capacity. J. Environ. Manag..

[36] Ek C., Miliute-Plepiene J. (2018). Behavioral spillovers from food-waste collection in Swedish municipalities. J. Environ. Econ. Manag..

[37] Whitmarsh L., O’Neill S. (2010). Green identity, green living? The role of pro-environmental self-identity in determining consistency across diverse pro-environmental behaviours. J. Environ. Psychol..

[38] Holbert R.L., Kwak N., Shah D.V. (2003). Environmental Concern, Patterns of Television Viewing, and Pro-Environmental Behaviors: Integrating Models of Media Consumption and Effects. J. Broadcast. Electron. Media.

[39] Hess S. (2014). Latent class structures: Taste heterogeneity and beyond. Handbook of Choice Modelling.

[40] Hawkins D.I., Best R.J., Coney K.A. (1998). Consumer Behavior: Building Marketing Strategy.

[41] Latacz-Lohmann U., Foster C. (1997). From ‘‘niche” to ‘‘mainstream”-strategies for marketing organic food in Germany and the UK. Br. Food J..

[42] Kaida N., Kaida K. (2015). Spillover effect of congestion charging on pro-environmental behavior. Environ. Dev. Sustain..

[43] Eby B., Carrico A.R., Truelove H.B. (2019). The influence of environmental identity labeling on the uptake of pro-environmental behaviors. Clim. Chang..

[44] Latinopoulos D., Mentis C., Bithas K. (2018). The impact of a public information campaign on preferences for marine environmental protection. The case of plastic waste. Mar. Pollut. Bull..

[45] Duerden M.D., Witt P.A. (2010). The impact of direct and indirect experiences on the development of environmental knowledge, attitudes, and behavior. J. Environ. Psychol..

[46] Telford R., Boote J.D., Cooper C.L. (2004). What does it mean to involve consumers successfully in NHS research? A consensus study. Health Expect..

[47] Klopčič M., Slokan P., Erjavec K. (2020). Consumer preference for nutrition and health claims: A multi-methodological approach. Food Qual. Prefer..

[48] Chekima B., Wafa S.A.W.S.K., Igau O.A., Chekima S., Sondoh S.L. (2016). Examining green consumerism motivational drivers: Does premium price and demographics matter to green purchasing?. J. Clean. Prod..

[49] Grasso S., Asioli D. (2020). Consumer preferences for upcycled ingredients: A case study with biscuits. Food Qual. Prefer..

[50] Nordlund A.M., Garvill J. (2002). Value structures behind pro-environmental behavior. Environ. Behav..

[51] Fujii S. (2006). Environmental concern, attitude toward frugality, and ease of behavior as determinants of pro-environmental behavior intentions. J. Environ. Psychol..

[52] Whitmarsh L. (2009). Behavioural responses to climate change: Asymmetry of intentions and impacts. J. Environ. Psychol..

[53] Korfiatis K.J., Hovardas T., Pantis J.D. (2003). Determinants of Environmental Behavior in Societies in Transition: Evidence from Five European Countries. Popul. Environ..

[54] Lindenberg S., Steg L. (2007). Normative, Gain and Hedonic Goal Frames Guiding Environmental Behavior. J. Soc. Issues.

[55] (2019). China Green Manure Research System. http://123.127.160.231/index.do?method=personal&userId=3074&product=021&productName.

[56] National Bureau of Statistics of China (2018). Urban Socio-Economic Survey Division. China City Statistical Yearbook 2018.

[57] Si H., Shi J.-G., Tang D., Wu G., Lan J. (2020). Understanding intention and behavior toward sustainable usage of bike sharing by extending the theory of planned behavior. Resour. Conserv. Recycl..

[58] Wang B., Hong G., Qin T., Fan W.-R., Yuan X.-C. (2019). Factors governing the willingness to pay for air pollution treatment: A case study in the Beijing-Tianjin-Hebei region. J. Clean. Prod..

[59] Ajzen I. (2002). Perceived Behavioral Control, Self-Efficacy, Locus of Control, and the Theory of Planned Behavior1. J. Appl. Soc. Psychol..

[60] Anderson J.C., Gerbing D.W. (1988). Structural Equation Modeling in Practice: A Review and Recommended Two-Step Approach. Psycho. Bull..

[61] Stenqvist C., Nilsson L.J. (2009). National Report on the Energy Efficiency Service Business in Sweden.

[62] Zaharah SF A., Rusli Y.M., Alias R. (2014). Consumers’ Response for Price Increment of Biodegradable Shopping Bags in Selected Hypermarkets in Selangor, Malaysia. Austra. J. Bas. Appl. Sci..

[63] Hair J.F., Sarstedt M., Ringle C.M., Mena J.A. (2012). An assessment of the use of partial least squares structural equation modeling in marketing research. J. Acad. Mark. Sci..

[64] Tarka P. (2018). An overview of structural equation modeling: Its beginnings, historical development, usefulness and controversies in the social sciences. Qual. Quant..

[65] Loebnitz N., Aschemann-Witzel J. (2016). Communicating organic food quality in China: Consumer perceptions of organic products and the effect of environmental value priming. Food Qual. Prefer..

[66] Schöll K., Markemann A., Zárate A.V. (2015). The Influence of Intervention Projects on Pig Production Marketing Groups in Vietnam. Agric. Agric. Sci. Procedia.

[67] Singh A., Verma P. (2017). Factors influencing Indian consumers’ actual buying behaviour towards organic food products. J. Clean. Prod..

[68] Ahmad I.A.H.H., Jinap S., Nasir M.S., Alias R., Muhammad A.K.S. (2012). Consumers’ demand and willingness to pay for rice attributes in Malaysia. Int. Food Res. J..

[69] Wang Y., Min Q., Han S. (2016). Understanding the effects of trust and risk on individual behavior toward social media platforms: A meta-analysis of the empirical evidence. Comput. Hum. Behav..

[70] Dirks K.T. (1999). The Effects of Interpersonal Trust on Work Group Performance. J. Appl. Psychol..

[71] Zsóka Á., Szerényi Z.M., Széchy A., Kocsis T. (2013). Greening due to environmental education? Environmental knowledge, attitudes, consumer behavior and everyday pro-environmental activities of Hungarian high school and university students. J. Clean. Prod..

[72] Pothitou M., Hanna R.F., Chalvatzis K. (2016). Environmental knowledge, pro-environmental behaviour and energy savings in households: An empirical study. Appl. Energy.

[73] Van der Werff E., Steg L., Keizer K. (2013). I am what I am, by looking past the present: The influence of biospheric values and past behavior on environmental self-identity. Environ. Behav..

[74] Abrahamse W., Steg L., Vlek C., Rothengatter T. (2005). A review of intervention studies aimed at household energy conservation. J. Environ. Psychol..

[75] Frick J., Kaiser F.G., Wilson M.R. (2004). Environmental knowledge and conservation behavior: Exploring prevalence and structure in a representative sample. Pers. Individ. Differ..

[76] Zareie B., Navimipour N.J. (2016). The impact of electronic environmental knowledge on the environmental behaviors of people. Comput. Hum. Behav..

[77] Wang X., Lin X., Spencer M.K. (2019). Exploring the effects of extrinsic motivation on consumer behaviors in social commerce: Revealing consumers’ perceptions of social commerce benefits. Int. J. Inf. Manag..

[78] Contini C., Boncinelli F., Marone E., Scozzafava G., Casini L. (2020). Drivers of plant-based convenience foods consumption: Results of a multicomponent extension of the theory of planned behaviour. Food Qual. Prefer..

[79] Jin H., Lin Z., McLeay F. (2020). Negative emotions, positive actions: Food safety and consumer intentions to purchase ethical food in China. Food Qual. Prefer..

